# Effect of cognitive training on episodic memory retrieval in amnestic mild cognitive impairment patients: study protocol for a clinical randomized controlled trial

**DOI:** 10.1186/s13063-018-3143-0

**Published:** 2019-01-08

**Authors:** Kan Zhang, Junyang Wang, Guoping Peng, Ping Liu, Fangping He, Zude Zhu, Benyan Luo

**Affiliations:** 1 Department of Neurology, The First Affiliated Hospital of Medical School of Zhejiang University, No.79 Qingchun Road, Hangzhou, 310003 China; 2 Collaborative Innovation Center for Language Competence, School of Linguistics and Arts, Jiangsu Normal University, Xuzhou, 221116 China; 3 Collaborative Innovation Center for Brain Science, Hangzhou, 310003 China

**Keywords:** Cognitive control training, MRI, Neural plasticity, aMCI, Clinical randomized trial

## Abstract

**Background:**

Mild cognitive impairment (MCI) is a transition state between asymptomatic stage and dementia. Amnestic MCI (aMCI) patients who mainly present with memory deficits are highly likely to progress to Alzheimer’s disease (AD). At present, no broadly effective drug therapy is available to prevent the progression from memory deficit to dementia. Cognitive control training, which has transfer effects on multiple cognitive capacities including memory function in healthy old adults, has not yet been applied to aMCI.

**Methods/Design:**

In this single-center, randomized double-blind placebo-controlled study, 70 aMCI patients will be recruited and randomly assigned to the training and control groups. The intervention is an Internet-based cognitive control training program performed for 30 min daily, five days per week, for 12 consecutive weeks. Neuropsychological assessment and structural and functional magnetic resonance imaging (MRI) will be performed at baseline and outcome. Primary outcomes are changes of episodic memory retrieval function. Secondary outcome measures are neuroplasticity changes measured by functional and structural MRI.

**Discussion:**

In this study, an Internet-based cognitive control training program is adopted to investigate whether cognitive control training can enhance the retrieval of episodic memory in aMCI patients. The combination of multi-modal MRI and neuropsychological tests could have a good sensitivity in evaluating the effects of cognitive control training and could also uncover the underlying neural underpinning.

**Trial registration:**

ClinicalTrials.gov, NCT03133052. Registered on 21 April 2017.

**Electronic supplementary material:**

The online version of this article (10.1186/s13063-018-3143-0) contains supplementary material, which is available to authorized users.

## Background

Alzheimer’s disease (AD) is the most common age-related neurodegenerative disorder. Cardinal AD symptoms are memory impairment and cognitive function decline. Mild cognitive impairment (MCI) refers to the transition state between the asymptomatic stage and dementia [1]. According to the affected cognitive domain, MCI is generally divided into amnestic MCI (aMCI) and non-amnestic MCI. aMCI is primarily manifested by memory deficits with or without other cognitive impairments, while non-amnestic MCI is mainly characterized by cognitive impairments other than memory deficits, such as decline in executive function, linguistic function, visuospatial function, and attention [2].

Approximately 10–15% of aMCI patients annually progress to AD and up to 80% progress to AD dementia within six years [3], although a proportion of this group never progress to develop dementia [4, 5]. Up to now there are no effective drug therapies available to prevent or slow this process. An efficacious non-medical approach is thus urgently required for MCI intervention.

Cognitive training is a promising non-drug alternative to slow MCI-to-AD progression. Studies show that game-style cognitive training can effectively improve the cognitive function of elderly individuals [6, 7] and even reverse the age-dependent decline in cognitive capacity. In general, recent meta-analyses further revealed promising training effect in MCI [8, 9] with comparable training improvement between MCI and healthy adults [8].

It remains unclear whether cognitive training is effective in promoting memory function, especially episodic memory function in aMCI. Episodic memory [10], a subtype of long-term memory, has the ability to remember past events as well as details about the context (e.g. times, places, persons). For instance, it enables us to form a detailed autobiographical event and represent our past experiences [11, 12]. Episodic memory consists of three distinct processes: encoding; storage; and retrieval. Encoding transforms the information of external stimuli or activates cognitive processes and aggregates the information. Followed by the encoding phase, information storage is triggered, which allows events to be maintained and recomposed into the long-term memory. Finally, retrieval processes are required to reactivate its mental representations and return the individual to his or her conscious experience of the event [13]. Episodic memory impairment is the earliest and most clinically significant neuropsychological manifestation of aMCI as well as an early predictor of progression to AD [3].

A common approach to enhance the memory function of the aMCI patients was mnemonic strategy training. Such training protocols, however, have yielded controversial results, as both positive [14, 15] and negative findings [16] were reported. Moreover, a meta-analysis found that memory intervention alone had limited efficacy for improving objective memory and other cognitive functions compared to the positive control group [17].

Another approach to enhance memory function is cognitive control training. Cognitive control refers to how an individual stores, plans, and controls relevant information according to task demands in the course of information processing. It involves coordinating the retrieval by working memory, inhibiting automatic retrieval, updating the retrieved strategy, attention control, and selection [18]. Cognitive control training aims to enhance an individual’s cognitive control abilities (e.g. sustained attention, working memory, and task switching) [19]. Several investigations [20–23] have found that cognitive control training can exhibit transfer effects on multiple cognitive capacities, even those not directly targeted by the specific training task, including short-term memory and delayed recall in healthy old adults [24–26]. Moreover, Carreti et al. [27] demonstrated that cognitive control training enhanced the long-term memory performance of MCI patients. Recent meta-analyses further suggest that cognitive control training such as working memory training is the most effective training in enhancing cognitive function across healthy and functional impaired older adults [8]. However, as most studies have combined different types of MCI, it remains unclear whether cognitive training can enhance the episodic memory in aMCI.

This trial is the first study to test the efficacy of cognitive control training on episodic memory retrieval function in aMCI patients using a double-blinded, randomized controlled trial design. A further character of the current trial is investigating the neural underpinning of the cognitive control training. While previous studies have shown neural plasticity including functional response and connectivity, and both gray and white matter anatomical change [19, 28–32], how the critical cognitive control related network compensate the memory function is unclear. Recent studies in healthy older adults have showed that older adults recruited the prefrontal cortex during episodic memory retrieval in compensating their low cognitive control capacity [33]. Nevertheless, the underlying processes of episodic memory (especially the retrieval phase) affected by cognitive control training have not been reported yet.

Taken together, the present trial will examine the efficacy of cognitive training on episodic memory of aMCI patients. If cognitive training is effective in improving episodic memory function, then greater improvement should be observed in the training group than control group. Moreover, functional and structural data would provide further neural plasticity information about the training-related improvement.

## Methods/Design

### Study design

**Fig. 1:**
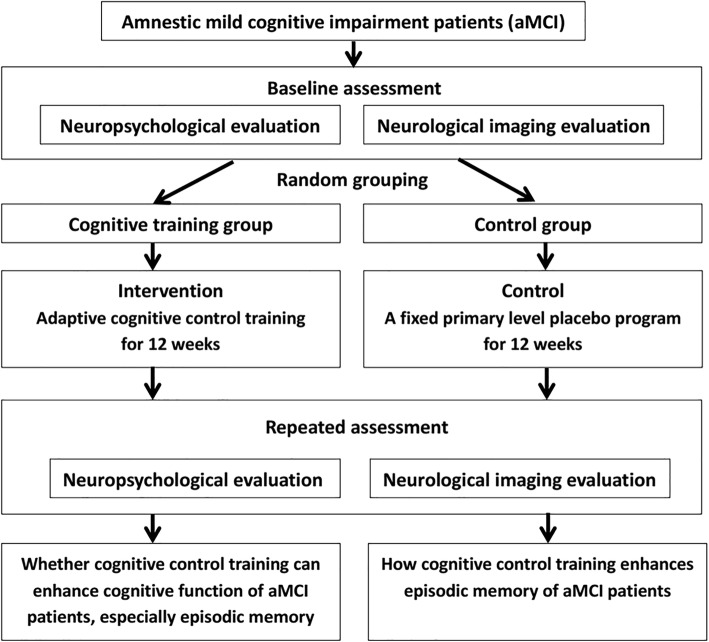
*Flow chart* of the experimental design

This is a single-center, randomized, double-blind, placebo-controlled trial registered at clinicaltrial.gov (NCT03133052). The study report will comply with the CONSORT statement and CONSORT-NPT (non-pharmacologic treatments) statement [34, 35]. The primary goal of this trial is to investigate whether cognitive control training can enhance episodic memory retrieval in aMCI patients. The secondary goal of this trial is to evaluate the efficacy of cognitive control training on neural plasticity in aMCI patients. The expected flow of patients through the trial can be seen in Fig. 1. The SPIRIT checklist with the recommended items to address in a clinical trial protocol is available (Additional file 1).

### Participants

Seventy qualified individuals are currently being recruited from the memory clinic of the First Affiliated Hospital of Zhejiang University and randomly assigned to the cognitive training or control group.

### Inclusion criteria

1. Age > 55 years;
2. Elementary school education or higher;
3. Chief complaint of memory impairment confirmed by relatives;
4. Ability to read a computer screen;
5. Normal overall cognitive function as evidenced by Clinical Dementia Rating-global (CDR-global) = 0.5, Mini-mental State Examination (MMSE) score ≥ 22 with a concurrent Montreal Cognitive Assessment (MoCA) score ≤ 20 for those with elementary educational level and MMSE score ≥ 26 with a MoCA score ≤ 25 for those with junior educational level or above;
6. Auditory Verbal Learning Test-Huashan version (AVLT-H) scores 1.5 standard deviations lower than age-matched controls;
7. Activities of Daily Living assessment-20 (ADL-20) ≤ 23;
8. Do not comply with AD diagnostic criteria proposed by the International Statistical Classification of Diseases and Related Health Problems, 10th Revision (ICD-10 for research use) and the National Institute of Neurological and Communicative Disorders and Stroke and Alzheimer’s Disease and Related Disorders Association (NINCDS-ADRDA).

### Exclusion criteria

1. Lack of a home computer or computer literacy;
2. A medical history of stroke and nervous system lesions, cerebral infarction, encephalomalacia lesions, or other space occupying lesions detected by head MRI plain scan;
3. Other nervous system diseases likely to cause brain dysfunction, such as schizophrenia, severe depression, frontotemporal lobe dementia, Huntington’s disease, brain tumors, Parkinson’s disease, metabolic encephalopathy, encephalitis, multiple sclerosis, epilepsy, brain trauma, or normal hydrocephalus;
4. Other systematic diseases likely to impair cognition, such as hypothyroidism, deficiency of folic acid or vitamin B12, or viral infection (syphilis or HIV);
5. Ethanol or drug misuse;
6. Severe liver and renal insufficiency, pulmonary incompetence, anemia, malnutrition, gastrointestinal tract disease, arrhythmia, or heart infarction in the previous six months;
7. Metal implantation or other MRI contraindications;
8. Aphasia, consciousness disorder, or other diseases precluding cognitive examination;
9. Use of drugs that affect cognitive function, such as sedatives, anti-anxiety drugs, nootropic drugs, sleeping pills, and cholinomimetic drugs.

### Randomization

All individuals are randomly and evenly assigned to the cognitive training or control group using the random number table method by an independent statistician using SAS software (SAS Institute, Inc., Cary, NC, USA). Written informed consent is obtained from all participants. After enrolment, the random and intervention number labels are sent to the memory clinic. If a participant needs emergency treatment, blinding will be broken, and the participant will then be managed as off-trial.

### Blinding

The participants, nurses, neuroimaging specialists, statisticians, and psychologists are all blind to group allocation. Data management, evaluation, and analysis will also be conducted blind to group membership.

### Intervention

Cognitive control training will be conducted using a computerized self-adaption training pattern (i.e. individually adjusted task difficulty to bring individuals to their performance maximum). Training tasks which were effectively used in previous studies will be adopted [36], including Flanker task [37], n-back working memory task [38], task switching [39], and Stroop task [40]. To guarantee adaptability to training difficulty, different levels of difficulty will be established as in previous studies with larger sample sizes. Briefly, at the beginning, each individual is assigned a task of similar difficulty. On each training day, the participants are required to complete five tasks three times, with 2 min for each training session (for a total daily test time of 30 min). For each task, the task difficulty will be advanced when the accuracy rate exceeds 80%. To adjust for individual variation in adaptability, the number of types of stimuli, the presentation probability of each type of stimuli, and the size and duration of a stimulus were systematically set. To keep a systematical setting, only one parameter will be adjusted; the remaining parameters will remain unchanged when the task difficulty is elevated. If the accuracy rate of a certain task exceeds 80% of normal counterparts, the task will be substituted for another of higher difficulty. The training program will be implemented five times weekly for 12 consecutive weeks.

In the control group, the training tasks include just the Flanker task and 1-back task. Importantly, a fixed, primary difficulty level will be set. The training tasks will be performed five times weekly, 30 min per session, for 12 consecutive weeks.

With the guidance of doctors, the initial training will be done in clinic and the rest of the training will be completed at home. The training will be supervised by an independent neurologist by telephone and the Internet.

### Primary outcome measures

The primary outcome measure is memory retrieval function assessed by delayed recall of Auditory Verbal Learning Test-Huashan version (AVLT-H). To be specific, participants were asked to study a list of 12 frequent and concrete words three times. Words were presented acoustically and free recall was measured in an immediate recall condition, a 5-min short delayed recall condition, and a 20-min long delayed recall condition.

### Secondary outcome measures

Secondary outcome measures include neuronal plasticity metrics obtained by MRI. Structural MRI using three-dimensional (3D)-T1 and diffusion tensor imaging (DTI) techniques will be adopted to investigate gray matter volume, white matter fiber integrity, and nerve fiber connectivity among cognition-related brain regions (prefrontal lobe and parietal lobe) and memory-related brain regions (hippocampus, medial temporal lobe, and prefrontal lobe) in participants before and after cognitive control training. Functional MRI (fMRI) will be used to reveal changes in the cerebral activation. Resting-fMRI and task-fMRI will be performed to statistically compare changes in regional function signals within episodic memory- and cognitive control-related brain regions (frontal, prefrontal, and parietal lobes), and changes in connectivity among implementation, memory and default networks, especially the prefrontal lobe-hippocampus function connection. For task-fMRI, a scan with an episodic memory task will be included.

### Data collection

Demographic data (gender, age, educational level, and occupation), medical and disease histories, and results of physical examinations, nervous system examinations, neuropsychological evaluations, and blood analyses are collected from consecutive patients according to exclusion criteria. Qualified individuals will receive structural and functional head MRI scans.

Routine blood tests will be performed for detection of blood cell counts, liver and kidney function, blood glucose levels, thyroid function, folic acid and vitamin b12 levels, and syphilis and HIV antibodies.

After 12 weeks of cognitive training, repeat physical examinations, nervous system examinations, neuropsychological evaluations, and structural and functional MRI will be performed (Fig. 1).

### Neuropsychological assessment

**Fig. 2:**
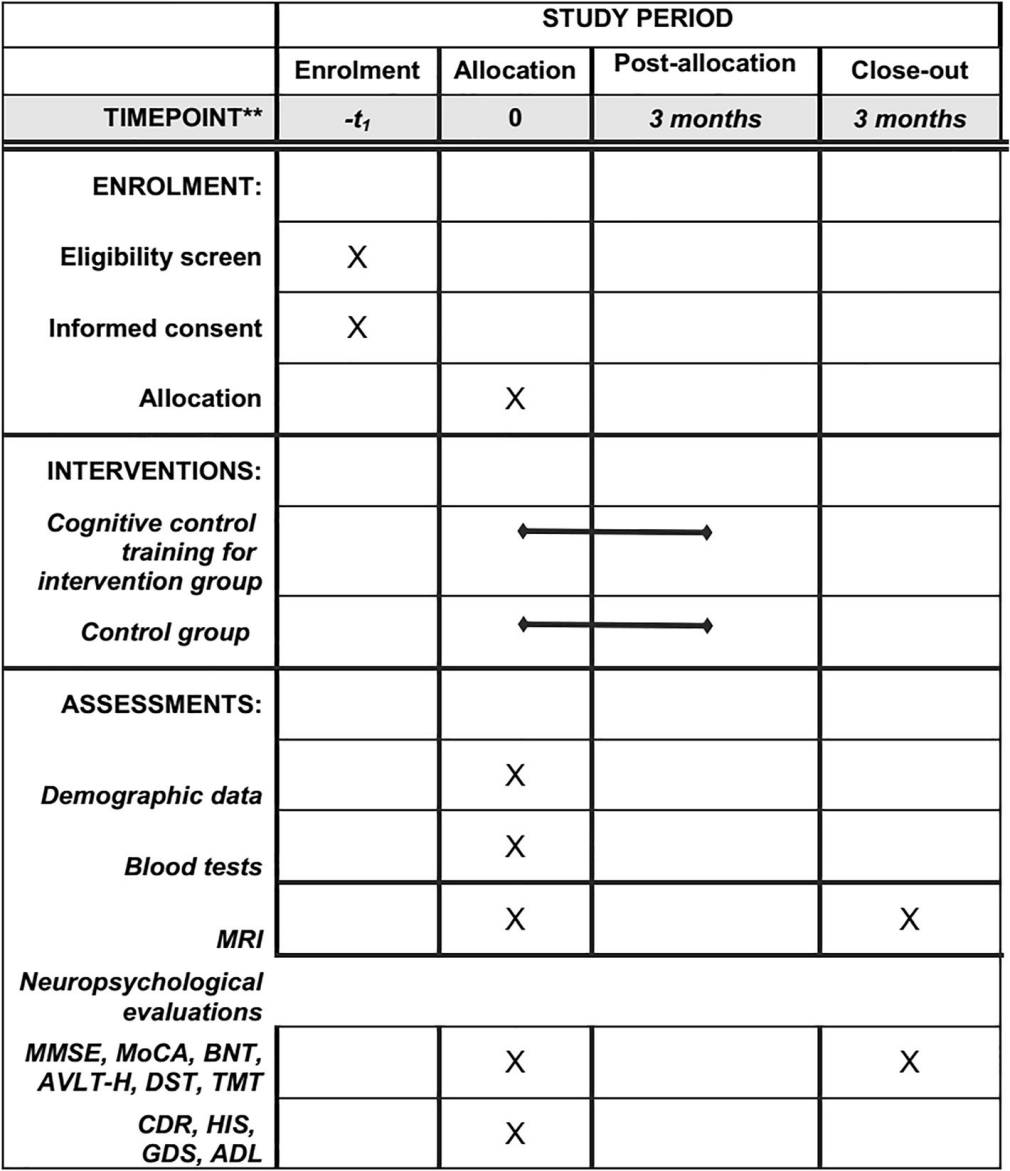
Standard Protocol Items: Recommendations for Interventional Trials (SPIRIT) figure with the schedule of enrollment, interventions and assessments. MRI magnetic resonance imaging, MMSE Mini-mental State Examination, MoCA Montreal Cognitive Assessment, BNT Boston Naming Test, AVLT-H Auditory Verbal Learning Test - Huashan version, DST DIGIT span test, TMT TRAIL Making Test, CDR Clinical Dementia Rating, HIS Hachinski Ischemia Scale, GDS Geriatric Depression Scale, ADL Activities of Daily Living

The neuropsychological assessment employs several commonly used measures of cognitive and daily functions. Measures include MMSE, MoCA, CDR, AVLT-H, Boston Naming Test (BNT), DIGIT span test (DST), TRAIL Making Test (TMT), Hachinski Ischemia Scale (HIS), Geriatric Depression Scale (GDS), and ADL assessment. Of these, CDR, HIS, GDS, and ADL will only be performed in the recruitment phase (Fig. 2). Furthermore, in the memory domain, episodic memory tasks that include a learning session and a test session will be conducted both outside and inside the scanner.

### MRI protocol

All participants will receive MRI scans at baseline and after 12 weeks of cognitive training using an identical 3-T device (GE DISCOVERY MR750) in the same scan modes. The 3D-T1 structural images of the brain will be obtained by 3D magnetization-prepared rapid acquisition with gradient echo (3D-MPRAGE) using the following acquisition parameters: 176 sagittal slices; thickness = 1 mm; TR = 8.2 ms; TE = 3.2 ms; flip angle = 8°; FOV = 250 × 250 mm; and matrix = 256 × 256. fMRI scan will be performed using a T2-weighted echo-planar imaging (EPI) sequence with the following acquisition parameters: slices = 43; TR = 2000 ms; TE = 30 ms; flip angle = 90°; FOV = 220 × 200 mm; and matrix = 64 × 64. DTI will be conducted using a single excitation EPI sequence and the following scan parameters: FOV = 192 × 192 mm; TR = 8600 ms; TE = 84 ms; b = 1000 s/mm^2^; diffusion sensitive gradient magnetic fields = 30; slices = 67; thickness = 1.5 mm; layer interval = 0 mm; NEX = 2; and 4 b0 images. Image quality will be validated by an experienced imaging specialist. Individuals with potential cerebral impairments or structural abnormalities revealed by neuroimaging will be excluded from subsequent experiments.

### Data monitoring

All neuropsychological evaluations will be conducted by the same neuropsychologist, all MRI scans completed using an identical machine, and image quality examined after each scan cycle.

### Sample size estimates

According to a previous study [41], in which computerized cognitive training was administered for six weeks, the mean difference in AVLT-H score between the cognitive training and control groups was 0.26 ± 0.3. Based upon this difference, 22 samples are required to yield a statistical power of 80% at a statistical significance level of 0.05. The sample size was estimated using the following equation:

$$ n=\frac{\left({q_1}^{-1}+{q_2}^{-1}\right){\left({t}_{\alpha /2}+{t}_{\beta}\right)}^2{S}^2}{\delta^2} $$

where q_1_ and q_2_ are the sample size ratio of two groups (q_1_ = q_2_ = 0.5), α is the statistical significance level (α = 0.05), β is calculated from 1-β which stands for statistical power (1-β = 0.8), δ is the mean difference (δ = 0.26), and S is the standard deviation (S = 0.3) used in the analyses. Considering the instability of standard deviation and dropout rate, the sample size for each group is set as 35.

### Adverse events

As far as we know, no adverse events caused by cognitive training have been reported yet.

### Statistical analysis

To make sure that baseline levels between groups (cognitive training, control) are comparable, questionnaire measures will be analyzed using independent sample t-tests. To determine the training efficacy from baseline to outcome, paired sample t-tests will be used in each group, respectively, for the changes in scores of trained tasks. The neuropsychological changes will be correlated with the score changes of the trained tasks to test the training effect on neuropsychological performance. To determine the training transfer effect, ANOVAs will then be performed on each dependent variable, with time (baseline, outcome) as the within-subjects factor and group (cognitive training, control) as the between-subjects factor.

## Discussion

For all we know, this is the first study to assess the effects of cognitive control training on episodic memory retrieval function in aMCI patients. In this study, first, we will examine the efficacy of cognitive control training on episodic memory of aMCI patients by using neuropsychological tests. We hypothesize that being immersed in a challenging, adaptive cognitive control training for a prolonged period of time (i.e. 12 weeks) would enhance cognitive control abilities as well as episodic memory function. Next, we will investigate the neural underpinning of the cognitive control training by conducting multimodal MRI. Accumulating studies have shown significant training-related brain state changes in healthy old seniors, specifically: (1) increases in gray matter volumes, particularly in the hippocampus [42]; (2) improved white matter integrity in the left uncinate fasciculus testified by an increase in fractional anisotropy [28]; and (3) activation of specific frontal and parietal cortical regions [38] and stronger connectivity in networks such as the default mode network and the central executive network [28, 43]. Thus, we hypothesize that the cognitive control training would result in similar effects on neural plasticity of older adults with aMCI.

There are several advantages in this study. First, the cognitive control training is Internet-based, allowing participants to accomplish the training at home conveniently and doctors to administer the training protocol and supervise training progress readily.

Second, task difficulty can be adaptive according to training performance (the better performance, the higher level of difficulty). This will reduce the effect of individual factors on training, thereby promoting participation and training effects.

Third, a positive control group accomplishing intervention tasks with constant task difficulty will be established to attenuate the placebo effect.

Finally, structural and functional MRI will be used to investigate underlying mechanisms. Traditionally, neuropsychological scales alone are employed to evaluate training efficacy. In this study, neuronal plasticity is combined with neuropsychological scales to reveal underlying neural mechanisms.

There are several limitations in this study. First, the test battery reported in this study is limited: in consideration of the individuals’ attention span and compliance, we do not include scales such as visual attention (e.g. Bells test, etc.), praxis (e.g. imitation of gestures, etc.), tests of reasoning (e.g. Raven matrices, etc.), verbal fluency (e.g. phonemic verbal fluency task, etc.), inhibition (e.g. Stroop test, etc.), and planning (e.g. Towers of London test, etc.). Second, the topic of this study is MCI due to AD. Theoretically, AD biomarkers should be performed (e.g. FDG-PET for mesial temporal glucose hypometabolism, PET-amyloid, etc.). However, it is inaccessible for every participant to perform AD biomarkers (e.g. low compliance and high expense). The diagnosis of MCI due to AD has to depend on clinical symptoms, neuropsychological tests, and MRI in this study.

One big challenge that we may meet for the study is the difficulty in maintaining participants. The aMCI patients who agree to participate in the study may decide to quit the program during the 12 consecutive weeks. Discontinuing from the intervention program may be attributed to personal issues (e.g. diseases, busyness, or low incentive) or family reasons (e.g. moving, need to care for their partner, or lack of caregiver’s supervision) that may impede them from completing the study. We calculated the dropout rate in the sample size estimation to make sure that we could reach adequate power for the study.

## Trial status

This trial commenced recruitment of aMCI patients in July 2017 and is expected to continue through January 2019. The trial is currently recruiting.

## Additional file

Additional file 1: SPIRIT 2013 Checklist: recommended items to address in a clinical trial protocol and related documents. (DOC 121 kb)

## Abbreviations

3D-MPRAGE: Three-dimensional (3D) magnetization-prepared rapid acquisition with gradient echo
AD: Alzheimer’s disease
ADL: Activities of Daily Living
aMCI: Amnestic mild cognitive impairment
AVLT-H: Auditory Verbal Learning Test-Huashan version
BNT: Boston Naming Test
CDR: Clinical Dementia Rating
CONSORT: Consolidated Standards of Reporting Trials
DTI: Diffusion tensor imaging
DTS: DIGIT span test
EPI: Echo-planar imaging
fMRI: Functional magnetic resonance imaging
GDS: Geriatric Depression Scale
HIS: Hachinski Ischemia Scale
ICD-10: International Statistical Classification of Diseases and Related Health Problems, 10th Revision
MCI: Mild cognitive impairment
MMSE: Mini-mental State Examination
MoCA: Montreal Cognitive Assessment
MRI: Magnetic resonance imaging
NINCDS-ADRDA: National Institute of Neurological and Communicative Disorders and Stroke and Alzheimer’s Disease and Related Disorders Association
TMT: TRAIL Making Test
